# Video‐endoscopic versus open inguinal lymphadenectomy: Long‐term oncological outcomes in penile cancer

**DOI:** 10.1002/bco2.70153

**Published:** 2026-01-06

**Authors:** Ranya Kumar, Krishna Sethia, Vivekanandan Kumar

**Affiliations:** ^1^ School of Clinical Medicine University of Cambridge Cambridge UK; ^2^ Norfolk and Norwich University Hospitals NHS Trust Norwich UK; ^3^ University of East Anglia Norwich UK

**Keywords:** inguinal lymph node dissection, nodal status, open ILND, penile cancer, survival, video‐endoscopic

## Abstract

**Introduction:**

Lymph node metastasis status is the strongest predictive factor for penile cancer survival. In penile cancer patients with suspected lymph node involvement, inguinal lymph node dissection (ILND) extends disease‐free survival. Though video‐endoscopic ILND (VEILND) has demonstrated superior surgical outcomes to open ILND (OILND) in the short term, its oncological efficacy long term is unproven. We present our long‐term oncological follow‐up of our previously published ILND cohort.

**Methods:**

A prospectively collected institutional database was used to determine the outcome in 42 consecutive patients treated for penile cancer in a tertiary referral centre between 2008 and 2015. Overall survival and cancer‐specific survival (CSS) were calculated using Kaplan–Meier curves and compared via log‐rank tests.

**Results:**

Forty‐two patients underwent 68 ILND (35 OILND vs. 33 VEILND). Thirteen out of 42 patients were alive at a mean follow‐up of 12.5 years. Overall survival for OILND and VEILND was 36.4% and 30.0% at 10 years. There was no significant difference between the survival curves (*p* = 0.91). CSS was equivalent (*p* = 0.87). Ten‐year CSS was 75.3% (OILND) and 65.5% (VEILND). When stratified by nodal status, CSS for OILND was 77.8%, 83.3%, 50% and 66.7% (N0, N1, N2 and N3) compared with VEILND which were 100%, 75%, 75% and 40% respectively at 8 years. Thus, there was no significant difference in CSS between patients undergoing VEILND and OILND stratified by nodal status. Moreover, inguinal or pelvic nodal recurrence rate was equivalent in both groups, occurring in 5/22 OILND and 4/20 VEILND (*p* = 1.00) patients.

**Conclusion:**

To our knowledge, we present the first European report of long‐term follow‐up demonstrating the oncological safety of VEILND. VEILND has comparable outcomes of recurrence, overall survival and CSS, with significantly reduced complication rates and length of stay, in penile cancer at a median follow‐up of 104 months (range 2–213 months).

## 1 INTRODUCTION

In the Western world, the incidence of penile cancer is significantly lower (0.4–1 per 100 000) than in the Eastern world (1.3 per 100 000).^1^, ^2^ Studies have shown that lymph node metastasis status is the strongest predictive factor for penile cancer survival.^3^ In patients with palpable inguinal nodes, 70% to 90% harbour metastatic disease. In such patients with suspected lymph node involvement, inguinal lymph node dissection (ILND) extends disease‐free survival.^4^

Open radical ILND is the standard of care for node clearance. However, it has a very high complication rate ranging from 30% to 75%.^5^ Several modifications have been practiced to reduce the complication rate including the use of modified radical^6^ and fascial sparing^7^ approaches for ILND. These techniques offer dissection of a smaller field, preservation of the saphenous vein and not transposing sartorius, thus reducing morbidity without compromising the lymph node yield. Notwithstanding, the complications are still significant.

Ten per cent to 30% of the patients with impalpable nodes have micrometastatic disease,^8^ early identification and treatment of which improves survival. Disease specific 3‐year survival of patients with positive lymph nodes detected during surveillance was 35% and, in those who underwent early resection, 84%.^9^

Current methods of ILND have a prohibitively high morbidity as a diagnostic procedure in this setting.^10^ Several alternative techniques have been described including lymph node sampling, modified superficial ILND and dynamic sentinel node biopsy (DSNB) for non‐palpable lymph nodes. Though large high‐volume European centres have achieved a low false negative rate (FNR) of 5%,^11^ FNR is higher at approximately 10% in other populations.^12^, ^13^ Further, the International Atomic Energy Agency, among others, has highlighted limited availability of radiopharmaceuticals, regulatory barriers and shortage of equipment and trained personnel as major barriers preventing DSNB's widespread adoption, particularly in low‐ and middle‐income countries.^14^, ^15^, ^16^ Thus, the development of a simple less resource‐intensive lymph node procedure with lower morbidity is of paramount importance to penile cancer patients' quality of life.

Following their introduction by Ott, Kelling and Jacobeus in 1901, minimally invasive procedures have revolutionised surgery, decreasing complications and improving outcomes compared with their open counterparts. However, uptake has been slow for penile cancer treatment/inguinal lymphadenectomy because of the rarity of the disease and the lack of sufficient experience in minimally invasive surgery among the andrologists who manage penile cancer.

Video‐endoscopic ILND (VEILND) has been developed as a minimally invasive alternative to open ILND (OILND) to reduce the complication rates^17^, ^18^ without compromising oncological outcome. VEILND has demonstrated superior surgical outcomes in length of stay, blood loss and wound complication rates compared with OILND in the short term.

Though VEILND appears a promising candidate in the short term, its oncological efficacy long‐term is completely unknown and is used as a criticism against wider adoption of this technique. European Association of Urology (EAU)–American Society of Clinical Oncology (ASCO) and European Society for Medical Oncology (ESMO) guidelines recommend the usage of VEILND only as part of a clinical trial.^19^ To advance the evidence base of penile cancer guidelines worldwide and thus to improve the lives of patients directly affected by these guidelines, longer term follow‐up of VEILND is necessary beyond that which is currently available. Hence, we present the long‐term oncological follow‐up over a 15‐year period of our previously published ILND cohort.

## 2 METHODS

Our department is a tertiary referral centre serving a population of three million and receiving referrals from nine regional urological units, genito‐urinary medicine and dermatology clinics.

### 2.1 Patient selection and preoperative assessment

All penile cancer patients with palpable lymph nodes underwent ultrasound of the groin and fine needle aspiration cytology of the nodes. If FNA was positive, they underwent ILND directly. Some patients with radiologically suspicious nodes—yet a negative FNA had ILND after informed consent. All clinically node‐negative (cN0) patients after negative ultrasound underwent DSNB. If the DSNB is positive, they had completion ILND. All patients have had a CT and/or MRI scan to exclude metastatic disease.

### 2.2 Surgical procedures

We performed OILND from 2008 to 2013. We introduced VEILND from 2013 onwards with the support of our supra‐network multidisciplinary team, and all procedures were done consecutively. There was no case selection involved. OILND and VEILND were performed with the same template, and the techniques were as described previously.^20^

### 2.3 Follow‐up

Pathologically inguinal node‐positive patients were restaged with CT chest, abdomen and pelvis and discussed in supra‐network MDT to consider pelvic lymph node dissection and/or adjuvant chemoradiotherapy. Adjuvant therapy was recommended on an individualised approach by the MDT based on the CT findings, renal function, comorbidities, patient fitness for further surgery and the patient's preference. During follow‐up, patients had three‐monthly clinical examinations in the first year, four‐monthly in the second year and six‐monthly thereafter for life as VEILND was a newer procedure when we started. Additionally, they were offered a CT scan every 6 months for 3 years and yearly thereafter. Pathologically node‐negative patients have had the same clinical follow‐up schedule with additional ultrasound groin every 6 months, instead of CT scan, for 3 years.

### 2.4 Data and statistical analysis

Comparison between groups was performed using the unpaired *t* test for numerical variables, with chi‐squared test and Fisher's exact test used for dichotomous and categorical variables. Overall survival and cancer‐specific survival (CSS) were calculated using Kaplan–Meier curves and compared via log‐rank test. Univariate and multivariate logistic regression analyses examined the impact of salient predictors of survival—namely, age, T stage, grade, pathological node status, lymphoedema status and access (OILND vs. VEILND).

## 3 RESULTS

The baseline data, surgical techniques and complications (as classified by the Clavien–Dindo classification) have been reported in a previous publication.^20^ VEILND was superior to OILND regarding length of stay and complication rate. However, VEILND is not widely accepted yet due to the paucity of comparative long‐term oncological data.^21^ In this paper, we report the retrospective analysis of the prospectively collected data regarding long‐term oncological outcomes.

Forty‐two patients underwent 68 ILND (35 OILND vs. 33 VEILND) over a 7‐year period. All 68 procedures were done in a consecutive fashion. VEILND yielded significantly more nodes than OILND (9.36 vs. 7.11, *p* = 0.013) and significantly more positive nodes (1.24 vs. 0.57, *p* = 0.03) (Table 1). Lymph node density (0.13 vs. 0.08) was similar in both groups (*p* = 0.13).

**TABLE 1:** Patient demographics and baseline characteristics in patients undergoing ILND for carcinoma of the penis.

Variable	OILND	VEILND	*p* value
Age (years)	69.5	65.8	NS
Number of patients	22	20	NS
Number of groins	35	33	NS
Side of surgery: left	20	18	NS
Side of surgery: right	15	15	NS
Mean operative time (min)	94	97	NS
Mean number of lymph nodes	7.11	9.37	0.013
Mean number of positive lymph nodes	0.57	1.24	0.03
Mean lymph node density	0.08	0.13	NS

The median follow‐up times for VEILND and OILND patients were 96 (mean 80, range 1–128) and 102 (mean 93, range 1–204) months, respectively (Table 2).

**TABLE 2:** Overall survival and cancer‐specific survival at various duration of follow‐up.

Duration of follow‐up	OILND OS No.	VEILND OS No.	OILND CSS No.	VEILND CSS No.	*p* value
2 years	15	16	18	17	NA
5 years	14	14	16	16	NA
10 years	8	8	16	15	NA

Duration of follow‐up	OILND OS %	VEILND OS%	OILND CSS %	VEILND CSS %	*p* value
2 years	68.2	80.0	80.7	90.0	0.870
5 years	63.6	70.0	75.3	84.0	0.438
10 years	36.4	30.0	75.3	65.5	0.607

**FIGURE 1:**
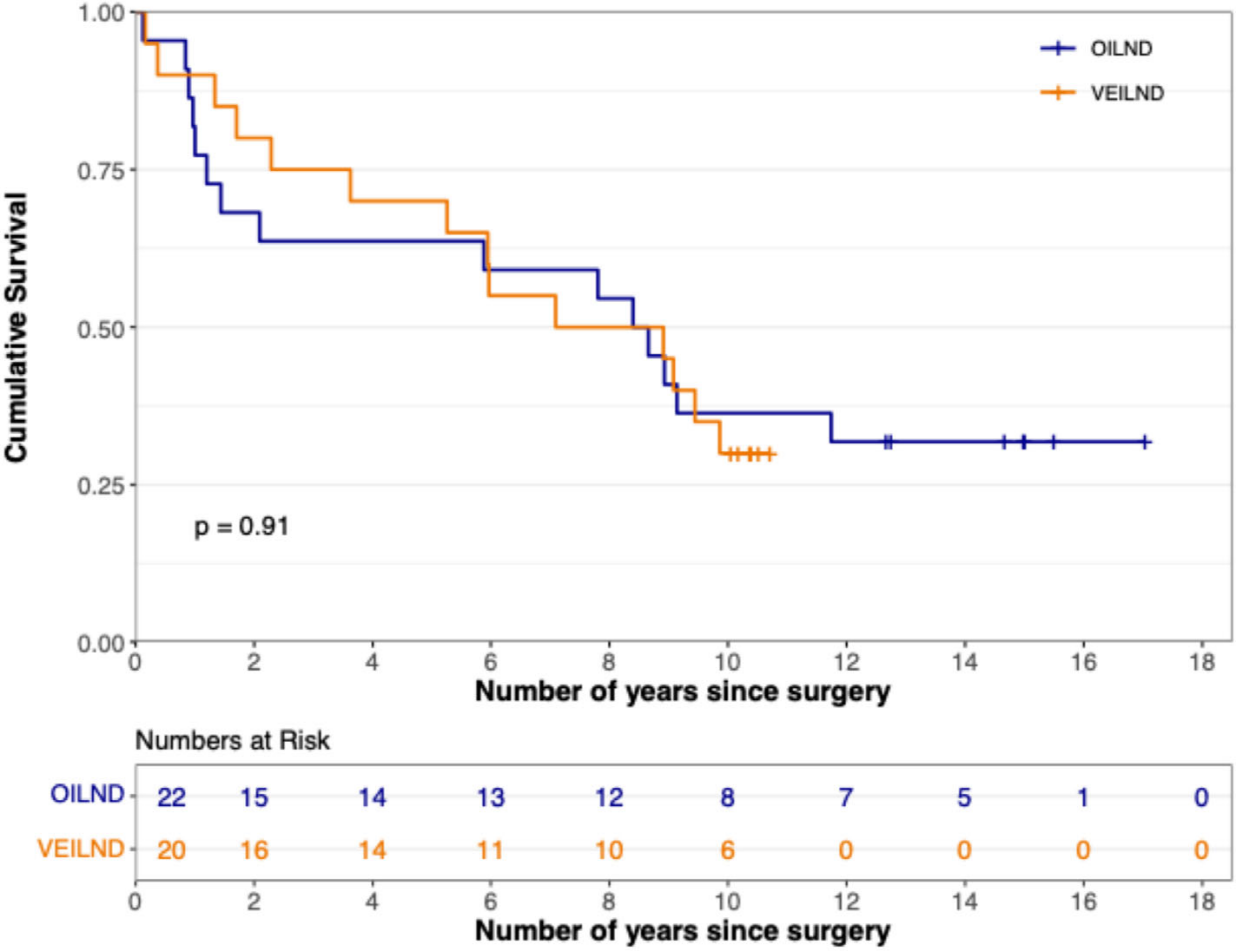
Kaplan–Meier curve of overall survival between OILND and VEILND patients.

**FIGURE 2:**
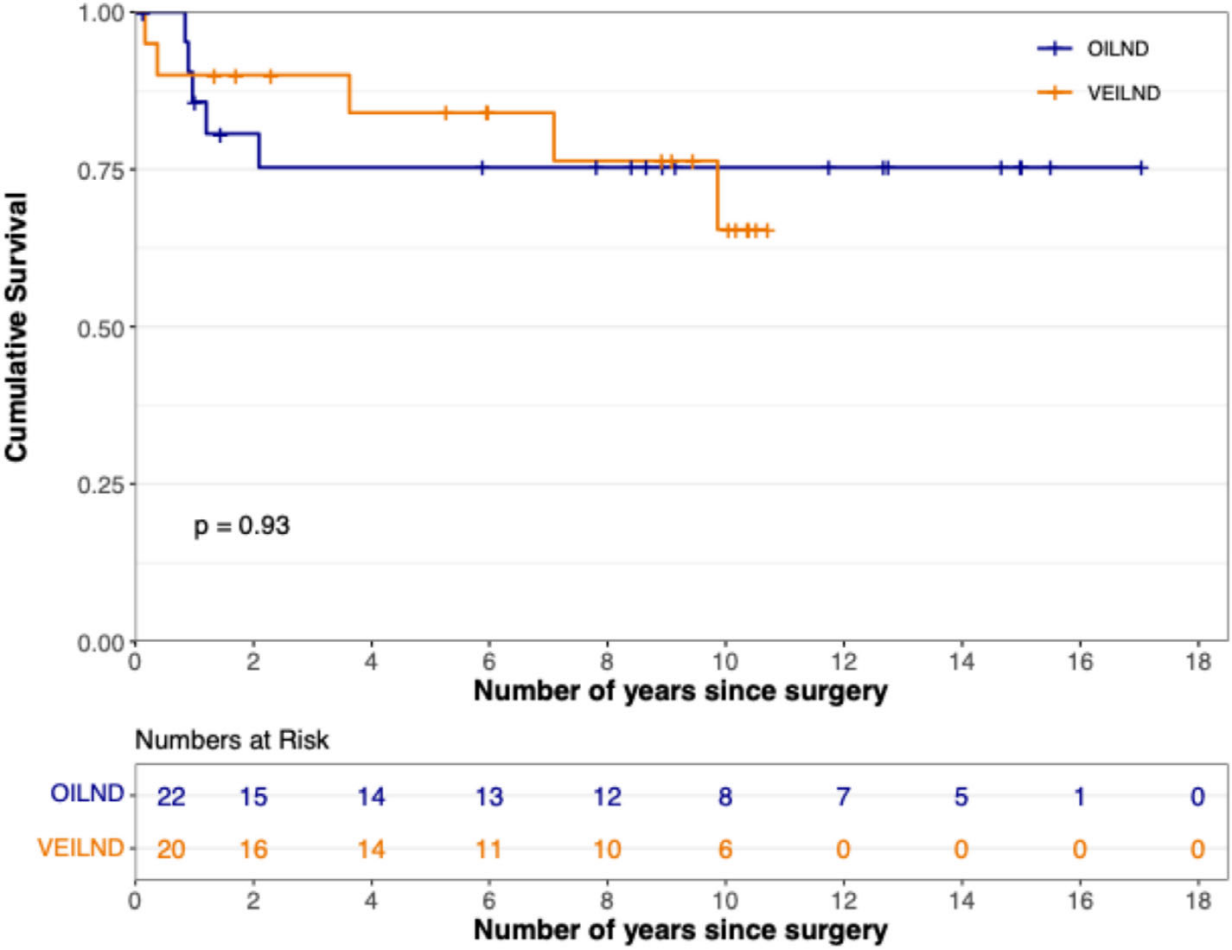
Kaplan–Meier curve of cancer‐specific survival between OILND and VEILND patients.

Thirteen out of 42 patients were alive at a median follow‐up of 8.5 years. There was no difference in the overall survival of VEILND and OILND patients (*p* = 0.91) (Figure 1). At 10 years, overall survival was 30.0% (VEILND) and 36.4% (OILND). Furthermore, CSS was equivalent (*p* = 0.93). Ten‐year CSS was 65.5% (VEILND) and 75.3% (OILND) (Figure 2). The median survival time was similar in both groups at 8.91 years for VEILND and 8.79 years for OILND. Inguinal or pelvic nodal recurrence occurred in four VEILND and five OILND patients (*p* = 0.83).

**FIGURE 3:**
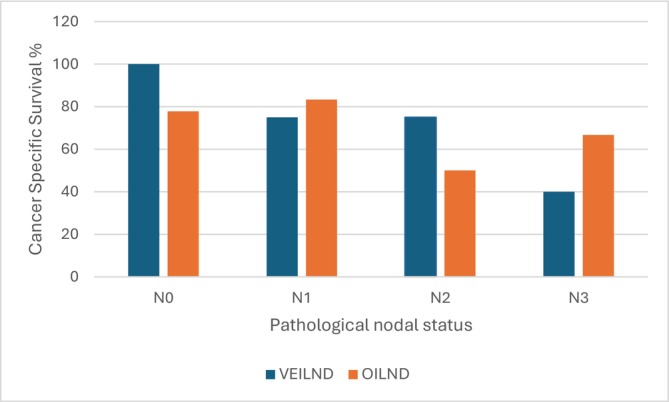
Percentage of patients surviving cancer in OILND and VEILND groups stratified by nodal status.

Node‐positive disease was present in 14 patients who had VEILND and 13 patients who had OILND (Table 3) was 63.5% and 72.9%, respectively, at 8 years (*p* = 0.59). When stratified by pathological nodal status (N0, N1, N2 and N3), the 8‐year CSS rates for OILND and VEILND were as follows (Figure 3): N0: 77.8% (OILND) versus 100% (VEILND) *p* = 0.23, N1: 83.3% (OILND) versus 75.0% (VEILND) *p* = 0.89, N2: 50% (OILND) versus 75% (VEILND) *p* = 0.69 and N3: 66.7% (OILND) versus 40% (VEILND) *p* = 0.51. We calculated 8‐year CSS as the 10‐year CSS is undefined as none are at risk at that time point for some groups.

**TABLE 3:** Distribution of pathological nodal status in OILND and VEILND groups.

Nodal status	OILND No.	VEILND No.	OILND %	VEILND %	*p* value
pN0	9	6	41	30	NS
pN1	7	5	32	25	NS
pN2	2	4	9	20	NS
pN3	4	5	18	25	NS
Total	22	20	100	100	

When considering age, T stage, grade, pathological node status, lymphoedema status and access, multivariate logistic regression showed only pN3 nodal status to be a significant predictor of CSS with *p* = 0.04. pN2 nodal status fell short of statistical significance with *p* = 0.06. VEILND versus OILND was not a predictor of survival in both univariate (*p* = 0.87) and multivariate regression (*p* = 0.88) analyses.

Adjuvant treatment was delivered to 15 out of 42 patients, seven of whom had undergone OILND (four adjuvant radiotherapy, two PLND and one chemotherapy) and eight of whom had undergone VEILND (four PLND, two adjuvant radiotherapy and one chemoradiotherapy).

Furthermore, two pN0 patients who had undergone OILND were diagnosed with metastatic disease during follow‐up, despite no inguinal or pelvic basin recurrence, whereas no VEILND pN0 patients died due to cancer‐specific causes. Both patients had locally advanced disease at diagnosis with total involvement of penis requiring total penectomy and perineal urethrostomy. Histologically, they were staged G3 pT3 disease with corporal involvement.

## 4 DISCUSSION

This is the first European study to report oncological outcomes of different techniques of ILND in penile cancer patients. Our previously published results of the same cohort clearly showed that VEILND is a safe technique, with reduced hospital stay and wound complication rate. Adoption of this technique is still low due to lack of long‐term oncological outcome data and no clear recommendation in the common urological guidelines. Our data suggest that VEILND has equivalent oncological outcomes to OILND at 2, 5 and 10 years, based on hard end points of overall survival and CSS. To our knowledge, this is the world's longest follow‐up reported comparing the two procedures in a large series of patients with penile cancer.^20^

Two recent systematic reviews^22^, ^23^ have been published looking at minimally invasive ILND (MIILND) which clearly identified a paucity of long‐term follow‐up data. The recent 2024 meta‐analysis identified three papers reporting 5‐year survival data. However, two of these papers included patients with a minimum follow‐up of less than 10 months. In particular, VEILND patients have shorter follow‐up compared with those who had OILND: Thyavihally et al. reported a median follow‐up of 42 months (i.e., <5 years) for the VEILND group, yet they reported a 5‐year survival of 65% and 66.8% for OILND and VEILND, respectively.^24^ Similarly, Shao et al. recorded a median follow‐up of 43 months.^25^ Thus, these estimates may be unstable due to censoring. Further, the aforementioned studies acknowledge non‐randomisation and had some form of selection bias. In contrast, in our study, at a median follow‐up of 104 months, both groups had comparable overall survival (32% vs. 30%) and CSS rates (75.3 vs. 65.5%).

There is a suggestion that VEILND may be a suitable alternative to OILND only as a diagnostic procedure for patients with early micro metastatic disease. Hence, it may not be suitable for cN2 or cN3 disease with larger nodes due to the risk of node fracturing. Thyavihally et al. have shown that, stratified by nodal status, there was no statistically significant difference in survival between OILND and VEILND group with 41 months follow‐up (*p* = 0.25).^24^ We have offered VEILND to all patients requiring ILND without selection bias, with a similar distribution of N2 and N3 patients across the two approaches (Table 3). Our study has also shown that there was no statistically significant difference in survival with a longer term follow‐up.

Surgery alone cannot cure N2/N3 disease, and adjuvant treatment is likely to improve survival in these high‐risk lymph node‐positive disease, though there is limited evidence.^26^, ^27^ All the node‐positive patients in our study were offered similar adjuvant treatment during follow‐up depending on their performance status and preference. There was equal distribution of adjuvant treatments in both groups; PLND and/or adjuvant radiotherapy was offered to all the pN3 patients who were fit for treatment.

EAU guidelines^28^ state that reported studies have a short follow‐up for MIILNDs and there was a high proportion of patients receiving VEILND or RAVEIL as prophylaxis as opposed to clinically node‐positive disease, precluding incorporation in the current guidance. In addition, some studies where OILND was used as a comparator involved more morbid manoeuvres when using an open approach, such as Sartorius transposition and saphenous vein sacrifice, which were not replicated in minimally invasive procedures. The distribution of N0 cases was similar in both groups with no prior case selection in either group. Moreover, we have not used sartorius transposition for any patient and the saphenous vein is preserved in both open and VEILND groups.

Further EAU–ASCO guidelines state that current evidence is very limited in cN1–cN2 patients and recommend minimally invasive approaches only as part of a clinical trial. ESMO guidelines^29^ state that the number of patients in the MIILND series is too small to conclude if there are any benefits in terms of oncological outcomes. Our study is one of the two^24^ largest comparative series so far and provides evidence across all nodal stages including N3 disease. Hence, the combined evidence demonstrated by our study and others confirms the suitable surgical and oncological outcomes of VEILND across a relatively large population with minimal confounders. Consequently, we advocate for the incorporation of VEILND into national guidelines to benefit patients through shorter hospital stays and fewer complications.

Two patients in the OILND group with N0 disease died of metastatic spread. Both of these patients have had T3 disease with corpora cavernosa involvement. In spite of total penectomy, these patients have had haematogenous spread of the disease without lymph node involvement. This is consistent with the observations of ourselves and Soria et al.,^3^ who have shown that T3 disease is an independent prognostic factor for survival in multivariate regression analysis.

This study is a non‐randomised case cohort study comparing the two ILND techniques. However, there is no selection bias involved as all the procedures were done consecutively with no prior case selection for either technique. Further, the baseline study population, patient characteristics, tumour characteristics and distribution of nodal staging were comparable in both groups. Considering the rarity of the disease and equivalence of outcome in both approaches in our study, it will require a large randomised multicentre study to settle the debate. Hence, a feasibility randomised study involving our group is underway at present to investigate the outcome between OILND and VEILND.^30^

One potential limitation of this study is the small cohort size, which may lack the statistical power to detect differences in survival. To our knowledge, no comparative studies have reported 10‐year survival outcomes in penile cancer.

Despite the small sample, our cohort demonstrated statistically significant differences in complication rates (6% vs. 68%) and length of hospital stay (2 vs. 7.5 days). Based on the observed mortality rate (70%) at 10 years, detecting a mortality difference of less than 33% would require a sample size of ~595 for sufficient power (*α* = 0.05, *β* = 0.20). Given the rarity of penile cancer, this is not feasible from any single‐centre currently.

The Penile Cancer Support Group at University College London Hospitals indicated that complications of the procedure was one of the top three most important factors that patients consider in relation to having lymphadenectomy for 9/9 (100%) of patients who completed the questionnaire. Given the severity of the disease, high baseline mortality and the impact of associated complications, our findings can serve as a valuable foundation for powering future randomised studies. Our group has recently completed recruitment of 50 patients for the Video‐endoscopic inguinal lymphadenectomy versus radical open dissection (VELRAD) trial, which aims to provide additional data necessary for more accurate power calculations. However, it will take approximately 10 years before meaningful long‐term outcomes from that trial will become available.

## 5 CONCLUSION

To our knowledge, we present the world's first report of long‐term follow‐up comparing survival in VEILND and OILND. VEILND has comparable outcomes of recurrence, median survival, overall survival and CSS in penile cancer at 10 years follow‐up. The only significant predictor of survival was the extent of nodal involvement, particularly pN3, rather than the surgical technique used. Further international randomised studies would be helpful to confirm these findings.

## AUTHOR CONTRIBUTIONS

*Conception and design*: Ranya Kumar, Krishna Sethia and Vivekanandan Kumar. *Acquisition of data*: Ranya Kumar and Vivekanandan Kumar. *Analysis and interpretation of data*: Ranya Kumar, Krishna Sethia and Vivekanandan Kumar. *Drafting of the manuscript*: Ranya Kumar and Vivekanandan Kumar. *Critical revision of the manuscript for important intellectual content*: Ranya Kumar, Krishna Sethia and Vivekanandan Kumar. *Statistical analysis*: Ranya Kumar and Vivekanandan Kumar. *Obtaining funding*: N/A. *Administrative, technical or material support*: Ranya Kumar. *Supervision*: Vivekanandan Kumar.

## CONFLICT OF INTEREST STATEMENT

The authors declare no conflicts of interest.
